# Influence of Release Parameters on Pitch Location in Skilled Baseball Pitching

**DOI:** 10.3389/fspor.2020.00036

**Published:** 2020-04-15

**Authors:** Ayane Kusafuka, Hirofumi Kobayashi, Takeshi Miki, Masumi Kuwata, Kazutoshi Kudo, Kimitaka Nakazawa, Shinji Wakao

**Affiliations:** ^1^Department of Life Science, Graduate School of Arts and Sciences, The University of Tokyo, Tokyo, Japan; ^2^Department of Science and Engineering, Waseda University, Tokyo, Japan

**Keywords:** baseball, pitch location, release parameter, accuracy, simulation

## Abstract

This study explored the mechanical factors that determine accuracy of a baseball pitching. In particular, we focused on the mechanical parameters at ball release, referred to as release parameters. The aim was to understand which parameter has the most deterministic influence on pitch location by measuring the release parameters during actual pitching and developing a simulation that predicts the pitch location from given release parameters. By comparing the fluctuation of the simulated pitch location when varying each release parameter, it was found that the elevation pitching angle and speed significantly influenced the vertical pitch location, and the azimuth pitching angle significantly influenced the horizontal pitch location. Moreover, a regression model was obtained to predict the pitch location, and it became clear that the significant predictors for the vertical pitch location were the elevation pitching angle, the speed, and spin axis, and those for the horizontal pitch location were the azimuth pitching angle, the spin axis, and horizontal release point. Therefore, it was suggested that the parameter most affecting pitch location weas pitching angle. On the other hand, multiple regression analyses revealed that the relation between release parameters varied between pitchers. The result is expected to contribute to an understanding of the mechanisms underlying accurate ball control skill in baseball pitching.

## Introduction

In various sport-related motor skills, such as throwing, kicking, and hitting, accurately controlling an object (typically a ball) to a target position is one of the most important skills. In this skill, there is a difference in performance, i.e., reproducibility of the ball arrival position, even among experts (Kawamura et al., 2017). The flight trajectory and final arrival position of the ball are physically determined by its state at the time of release or impact. For example, in baseball pitching, these are determined by the combination of nine mechanical ball parameters at release, referred to as release parameters. If the state at the time of release is always the same, the ball will always arrive at the same position. Therefore, the accuracy of final pitch location of the ball can be expected to be improved by increasing the reproducibility of release movements as much as possible. However, it is known that there is always variability in the release parameters, even for skilled players (Faisal et al., 2008).

It is also known that the choice of values of release parameters varies widely between different pitchers, even if the same position is targeted (Jinji and Sakurai, 2006; Nagami et al., 2011). As there are countless combinations of values of release parameters that achieve the same pitch location, i.e., there is redundancy, if a combination satisfies the desired conditions, then the pitcher can select various values of release parameters. However, considering the variability of release parameters, not all combinations can be considered equivalent.

The relationship between parameters influencing the results of movements have been evaluated in previous studies. The relation in which each parameter does not vary independently each time, but fluctuates while maintaining a relationship that compensates for the variability of other parameters has been reported. This is considered to be the best method, which is used by some skilled players, for improving accuracy of the arrival position without increasing reproducibility. The existence of such “compensatory coordination” which contributes to the stability of performance has been reported using many constrained virtual tasks (Müller and Loosch, 1999; Scholz and Schöner, 1999; Cohen and Sternad, 2012) and two-dimensional movements (relatively simple, not including torsion) (Kudo et al., 2000; Nasu et al., 2014).

Some sports skills, such as baseball pitching, involve more parameters for determining the arrival position than the number used in previous studies. In baseball pitching, it has been shown that the spin rate, which is the number of times the ball rotates per unit time, and the spin axis, which is the orientation of the ball rotation, differs between pitchers, and contribute to the ball trajectory in addition to the release position and release velocity vector (Jinji and Sakurai, 2006; Nagami et al., 2011). Considering that there is variability in each parameter and control of all parameters is necessary to realize accurate pitching, a question arises about the weighting; that is, which of the parameters is most dominant in determining pitch location. When more parameters are involved in the pitch location, their contribution to the pitch location may vary depending on their values. However, in previous studies, only a few parameters have been considered in combination, and it is not clear which parameter has the greatest influence on pitch location. Therefore, it is considered necessary to investigate the parameters independently first before considering their combination.

The purpose of this study was to investigate the degrees of influence of each baseball pitching release parameter on pitch location. In addition to the release point and the velocity vector used in the previous studies that performed the throwing task (Kudo et al., 2000; Nasu et al., 2014), the spin rate and the spin axis are important in pitching (Jinji and Sakurai, 2006; Nagami et al., 2011), and were used as release parameters. We created a simulation for pitch location prediction based on the release parameters and calculated the pitch location when each parameter is changed individually in actual examined ranges of baseball pitching. Comparing the magnitude of the fluctuation, it became clear which parameter influences the pitch location most. In previous studies, to find the most important kinematic variables that contribute to achieving a greater throw distance, regression prediction equations were used (Uday, 2019). Therefore, in addition to simulation analysis, in this study a regression model was obtained to predict the pitch location.

## Materials and Methods

### Measuring Data

Seven skilled baseball pitchers participated (sex: male; age: 28.1 ± 9.9 years; height: 175.9 ± 5.1 cm; body mass: 76.5 ± 3.5 kg; 6 right-handed and 1 left-handed), including one former professional pitcher from the NPB (Nippon Professional Baseball; Japan's top baseball league). They pitched 30 fastballs on the mound in an indoor stadium and were instructed to aim at the catcher's mitt, which was 90 cm above the ground, 40 cm outside from the center of home base (outside is defined based on the same side batters, e.g., a right-handed batter in case of a right-handed pitcher), and 50 cm behind home base. The data of 187 pitches were obtained, excluding data in which measurement errors occurred. The release parameters were measured using TrackMan Baseball (TRACKMAN). TrackMan Baseball detected release timing and the release parameters were taken. For the measurement of pitch location, a DV camera (Panasonic HC-V 100 M, Japan) was installed 7–8 m in front of home base, and the moment when the catcher caught the ball was photographed from the front side of the catcher (30 Hz). To obtain the position coordinates, we calibrated 3 points in the horizontal direction (1.5 m intervals) and 5 points in the vertical direction (0.5 m intervals) on the plane, giving a total of 15 calibration points for the catching position. The calibration points were digitized using numerical analysis software (MATLAB, Mathworks, Japan), and the average standard error was set to 1.0 cm or less. The position coordinates of the pitch location were calculated by digitizing the center point of the ball at the moment of catching and by using direct linear transformation. All participants provided informed consent, and the study was performed in accordance with the Declaration of Helsinki and with the approval of the ethics committee of the University of Tokyo.

### Simulation Analysis

**Figure 1:**
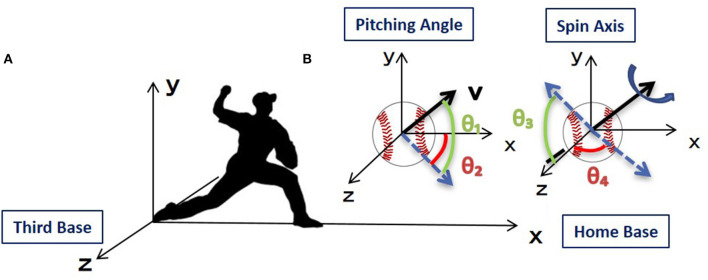
Three-dimensional orthogonal coordinate setup in this study. **(A)** The x axis runs from the mound toward home base, the y axis runs vertically upwards from the ground, and the z axis runs from the first base to the third base. The origin is the center of the plate on the mound. **(B)** The pitching angle is shown by the elevation angle θ_1_ (ranging from −90° to 90°) and azimuth angle θ_2_ (ranging from −90° to 90°), and the spin axis is shown by the elevation angle θ_3_ (ranging from −90° to 90°) and the azimuth angle θ_4_ (ranging from −90° to 90°).

To create a simulation for pitch location prediction, it was necessary to consider the mechanical elements acting on the ball at release. The three-dimensional orthogonal coordinates were defined as follows; the origin was taken as the center of the pitcher plate on the mound, the orientation of the x axis was in the direction of home base, the y axis was oriented vertically upwards, and the z axis was oriented in the third base direction (Figure 1A). Release parameters were defined based on this coordinate system. The pitching angle of the ball was given by the elevation angle θ1 (−90° to 90°) and the azimuth angle θ2 (−90° to 90°) in polar coordinates, and the rotation axis angle of the ball was given by the elevation angle θ3 (−90° to 90°) and azimuth angle θ4 (−180° to 180°) (Figure 1B). θ1 was the angle between the projection of the velocity on the x-y plane and the x-axis, and θ2 was the angle between the projection of the velocity on the x-z plane and the x-axis. θ3 was the angle between the projection of the spin axis on the y-z plane and the z-axis, and θ4 was the angle between the projection of the spin axis on the x-z plane and the z-axis. The positive direction of θ1 and θ3 was defined as upward direction, the positive direction of θ2 was defined as the direction of third base, and the positive direction of θ4 was defined as forward direction. Speed was defined as the magnitude of velocity vector, and spin rate was defined as the number of times the ball rotates per unit time.

Next, an equation of motion was set based on the coordinate axes, as follows. Generally, drag (F_d_) and lift (F_l_) are expressed as follows.

(1) $$\{\begin{array}{c} {\text{F}}_{\text{d}}=\frac{1}{2}\text{ρ}\text{S}{\text{V}}^{2}{\text{C}}_{\text{d}}\\ {\text{F}}_{\text{l}}=\frac{1}{2}\text{ρ}\text{S}{\text{V}}^{2}{\text{C}}_{\text{l}} \end{array}$$

where ρ (= 1.2 kg/m^3^) represents air density, S (= 4.3 × 10^−3^ m^2^) is the sectional area of the ball, and V is the velocity magnitude. C_d_ (= 0.35) and C_l_ (= (πS) 0.5 × spin rate/2) are the drag coefficient and lift coefficient, respectively, which are the same as those reported in (Kray et al., 2012).

When the spin axis of the ball is perpendicular to the traveling direction, the drag increases as the speed of the ball increases, and the lift increases as the rotation speed increases. When the spin axis is not perpendicular to the traveling direction, the lift force is perpendicular to both the traveling direction and spin axis, which means the lift force can be represented by the cross product of the traveling direction and spin axis of the ball. From the above discussion, the equation of motion of the ball considering drag and lift can be expressed as follows.

(2) $$\text{m}\left[\begin{array}{c} \frac{{\text{d}}^{2}\text{x}}{\text{d}{\text{t}}^{2}}\\ \frac{{\text{d}}^{2}\text{y}}{\text{d}{\text{t}}^{2}}\\ \frac{{\text{d}}^{2}\text{z}}{\text{d}{\text{t}}^{2}} \end{array}\right]=\frac{1}{2}\text{ρ}\text{SV}\left(-\text{Cd}\left[\begin{array}{c} \frac{\text{dx}}{\text{dt}}\\ \frac{\text{dy}}{\text{dt}}\\ \frac{\text{dz}}{\text{dt}} \end{array}\right]+\text{Cl}\frac{\text{H}}{\text{V}}\left[\begin{array}{c} \frac{\text{dx}}{\text{dt}}\\ \frac{\text{dy}}{\text{dt}}\\ \frac{\text{dz}}{\text{dt}} \end{array}\right]\times \left[\begin{array}{c} \text{ax}\\ \text{ay}\\ \text{az} \end{array}\right]\right)+\left[\begin{array}{c} 0\\ -\text{mg}\\ 0 \end{array}\right]$$

where m (= 0.145 kg) is the mass of the ball, H is the magnitude of the cross product of speed and spin axis, and g (= 9.81 m/s^2^) is gravitational acceleration.

a_x_, a_y_, and a_z_ are the rotational shaft angles of the ball as follows:

(3) $$\{\begin{array}{c} {\text{a}}_{\text{x}}=\text{cos }\theta 3\text{ sin}\theta 4\\ {\text{a}}_{\text{y}}=\text{sin }\theta 3\;\\ {\text{a}}_{\text{z}}=\text{cos }\theta 3\text{ cos}\theta 4 \end{array}$$

At this time, the equation of motion can be viewed as a second order differential equation with time t as a variable. The pitch location of the ball can be calculated by solving this equation of motion as an initial value problem of a differential equation. However, it is not possible to solve the equation algebraically if specific functions, such as the pitching trajectory, are not determined. Therefore, it is necessary to approximate the pitching trajectory through a numerical analysis. In this study, the Dormand–Prince method (Dormand and Prince, 1986) was applied. The specific calculation formula used in this study was the same as that used in (Kimura, 2009). By using this numerical analysis method, the change in position of the ball was calculated at every moment, and the calculation was repeated until the ball reached the catcher. To summarize the above, the following release parameters are used as initial conditions: release point (x, y, z), ball speed (v), pitching angle (θ1, θ_2_), spin rate (n), and spin axis (θ3, θ4). The pitch location (y, z), which was 50 cm behind home base in this study, is calculated based on these parameters and numerical analysis. Due to the limited functionality of TrackMan Baseball, it was not possible to measure the horizontal spin axis (θ4); thus, its simulated value was set to a constant of 30° because several studies (Jinji and Sakurai, 2006; Nagami et al., 2011) have shown that the mean values of the horizontal spin axis are 26–33°.

The variation in pitch location was simulated while varying each parameter individually. Each parameter was varied from its minimum to the maximum value for each pitcher, and the other parameters were fixed to the average for each pitcher. The results indicated that the larger the variation in the pitch location is, the higher the possibility that the pitch location is changed by the parameter.

### Multiple Regression Analysis

In addition to simulation analysis, a regression model was obtained to predict the pitch location. By backward-forward stepwise multiple regression analysis, the explanatory rate of the pitch location of each release parameter was calculated. This method finds the optimal combination of explanatory variables by reducing the number of explanatory variables from the most complex model (using all explanatory variables). If there is parameter whose p value is larger than 0.05, the parameter was reduced from the model. The regression was run separately for each pitcher. We used MATLAB to find a regression model with a coefficient of determination as close to 1 as possible.

## Results

### Variation in Pitch Location When Release Parameters Are Changed

The mean speed of the ball in this study was 32.6 ± 2.2 m/s, whereas it was 33.8 ± 1.7 m/s in Jinji and Sakurai (2006) and 37.7 ± 1.2 m/s in Nagami et al. (2011). The mean spin rate was 29.0 ± 2.8 rps, whereas it was 31.4 ± 2.7 rps in Jinji and Sakurai (2006) and 34.3 ± 3.5 m/s in Nagami et al. (2011). Although these parameters used in this study were slightly less than in those previous studies, they were within standard ranges and this suggests that the participants successfully performed baseball pitching and data was successfully obtained. The mean release parameters for each participant are summarized in Table 1. The average error of the measured pitch location and the simulated results using the measured release parameters was 5.8 ± 1.4 cm.

**Table 1:** Mean release parameters and pitch location for each pitcher.

**Pitcher**	**V**	**θ1**	**θ_2_**	**N**	**θ3**	**X**	**Y**	**Z**	**Pitch location Y**	**Pitch location Z**
	**[m/s]**	**[deg]**	**[deg]**	**[rps]**	**[deg]**	**[m]**	**[m]**	**[m]**	**[m]**	**[m]**
A	32.2 ± 1.0	1.38 ± 1.1	−3.66 ± 1.09	26.9 ± 2.3	−53.0 ± 3.8	1.72 ± 0.039	1.49 ± 0.025	0.25 ± 0.041	0.63 ± 0.30	−0.23 ± 0.20
B	33.0 ± 0.50	−1.27 ± 0.75	−3.81 ± 0.47	31.3 ± 0.87	−31.3 ± 3.7	1.73 ± 0.047	1.79 ± 0.029	0.50 ± 0.024	0.50 ± 0.25	−0.16 ± 0.11
C	33.4 ± 0.34	−0.72 ± 0.69	−2.70 ± 0.56	31.1 ± 0.64	−21.0 ± 2.9	1.69 ± 0.024	1.62 ± 0.022	0.38 ± 0.020	0.51 ± 0.21	−0.16 ± 0.14
D	33.2 ± 0.72	−0.26 ± 0.83	−4.00 ± 0.55	29.9 ± 1.0	−32.4 ± 5.3	1.73 ± 0.029	1.58 ± 0.012	0.48 ± 0.027	0.62 ± 0.26	−0.27 ± 0.13
E	35.9 ± 0.38	−0.49 ± 1.51	−3.85 ± 0.91	33.4 ± 1.1	−41.3 ± 3.9	1.63 ± 0.030	1.60 ± 0.019	0.58 ± 0.033	0.74 ± 0.43	0.048 ± 0.21
F	32.5 ± 0.49	1.50 ± 0.99	−3.38 ± 1.07	27.3 ± 4.7	−9.47 ± 5.9	1.78 ± 0.049	1.38 ± 0.022	0.81 ± 0.034	0.99 ± 0.26	−0.085 ± 0.35
G	28.0 ± 0.27	1.36 ± 1.64	−1.52 ± 0.61	24.6 ± 0.80	2.39 ± 6.1	1.81 ± 0.021	1.59 ± 0.001	0.15 ± 0.019	0.66 ± 0.44	−0.078 ± 0.21
Mean	32.6 ± 2.2	0.28 ± 0.99	−3.11 ± 0.80	29.0 ± 2.76	−25.2 ± 17.4	1.72 ± 0.06	1.55 ± 0.08	0.43 ± 0.20	0.66 ± 0.15	−0.17 ± 0.11

**Figure 2:**
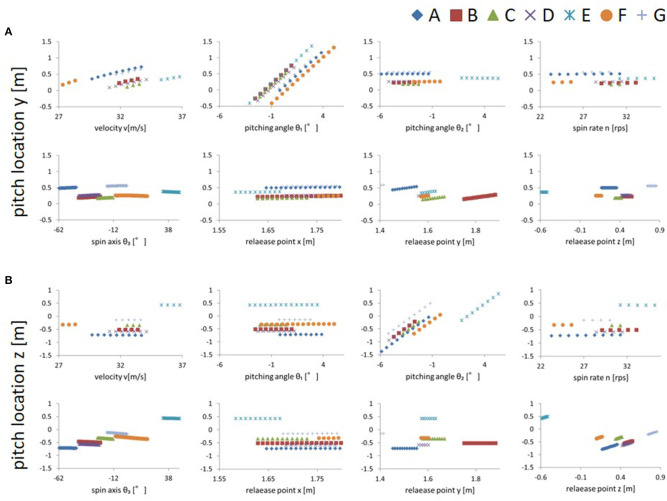
**(A)** Relationships between each parameter and vertical pitch location. The range of the vertical axis indicates the size of variation of the pitch location when each parameter is varied (other parameters were fixed at their mean values). **(B)** Relationship between each parameter and horizontal pitch location. The range of the vertical axis indicates the amount of variation of pitch location when each parameter is varied (other parameters were fixed at their mean values). Subject E was the only left-handed pitcher.

Comparing the variation in the pitch location caused by changing each release parameter, when the elevation pitching angle (θ1) was changed, the fluctuation of the vertical pitch location was the largest. Figure 2A shows the magnitude of the variation in vertical pitch location when changing the various mechanical parameters. The pitch location varied by approximately 30 cm each time the elevation pitching angle was changed by 1° and by ~20 cm each time the speed was changed by 1 m/s (= 3.6 km/h). In addition, the pitch location varied by 1 cm when the vertical release point was changed by 1 cm. The pitch locations for the other parameters only varied by a few centimeters, even when changed from the minimum to maximum value.

When the azimuth pitching angle (θ2) was changed, the fluctuation of the horizontal pitch location was the largest. Figure 2B shows the magnitude of variation in the horizontal pitch location when changing various release parameters. The pitch location varied by ~30 cm each time the azimuth pitching angle changed by 1°. In addition, the pitch location varied by 1 cm when the horizontal release point changed by 1 cm. The pitch locations for the other parameters only varied by a few centimeters, even when changed from the minimum to maximum value.

**Figure 3:**
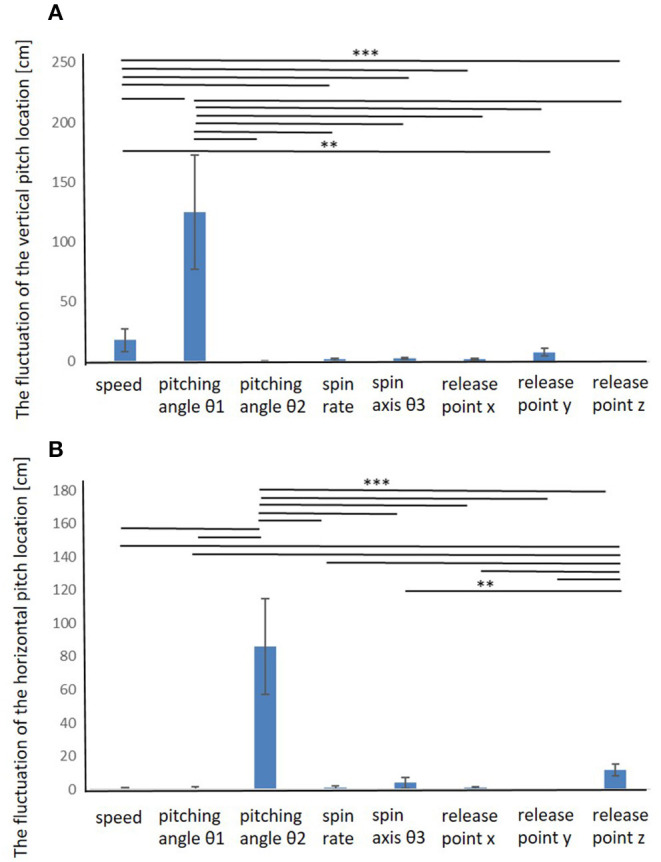
Fluctuation of the vertical and horizontal pitch locations when each release parameter was changed. The elevation pitching angle (θ_1_) and speed significantly influenced the vertical pitch location, and the azimuth pitching angle (θ_2_) significantly influenced the horizontal pitch location. **0.01≤ *p* <0.05, ***p < 0.01.

The fluctuation of the vertical and horizontal pitch location is summarized in Figure 3 (cf. Tables S1, S2). It was shown that the elevation pitching angle (θ1) and speed significantly influenced the vertical pitch location, and the azimuth pitching angle (θ2) significantly influenced the horizontal pitch location. The results had a similar trend among all pitchers.

### Regression Model to Predict Pitch Location

**Figure 4:**
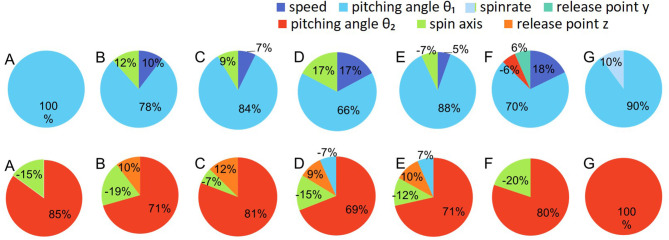
Predictive parameters of vertical (top row) and horizontal (bottom row) pitch locations for each pitcher. The values indicate the *t* value of each parameter. The explanatory rate of each parameter was different for each pitcher.

Multiple regression was used with the release parameters to independently establish regression equations for each pitcher. The average *R*^2^ values of regression models to predict the vertical pitch and horizontal pitch location for each pitcher were 0.97 ± 0.02 and 0.96 ± 0.04, respectively. Tables 2A,B list the regression coefficients of the significant predictors (*p* < 0.01) for each pitcher in vertical and horizontal pitch location, respectively (cf. Table S3). The results indicated that the significant predictors among all pitchers are the elevation pitching angle (θ1) for the vertical pitch location and the azimuth pitching angle (θ2) for the horizontal pitch location. For most of the pitchers, the speed and spin axis (θ3) were the significant predictors for the vertical pitch location, and the spin axis (θ3) and horizontal release point were the significant predictors for the horizontal pitch location. The explanatory rate of each parameter was different for each pitcher (Figure 4).

**Table 2:** Coefficient of regression equation for each patient.

**Pitcher**	**v**	**θ1**	**θ_2_**	***n***	**θ3**	***x***	***y***	***z***	**R^**∧**^2**
**(A)**									
A	–	0.195	–	–	–	–	–	–	0.945
B	0.054	0.318	–	–	0.009	–	–	–	0.988
C	0.060	0.315	–	–	0.008	–	–	–	0.978
D	0.100	0.322	–	–	0.014	–	–	–	0.986
E	0.061	0.300	–	–	−0.009	–	–	–	0.991
F	0.143	0.298	−0.024	–	–	–	0.988	–	0.984
G	–	0.235	–	0.054	–	–	–	–	0.931
Mean									0.97 ± 0.02
**(B)**									
**Pitcher**	**v**	**θ1**	**θ_2_**	***n***	**θ3**	***x***	***y***	***z***	**R**^**∧**^**2**
A	–	–	0.173	–	−0.009	–	–	–	0.911
B	–	–	0.215	–	−0.007	–	–	0.624	0.988
C	–	–	0.225	–	−0.004	–	–	1.164	0.992
D	–	−0.015	0.223	–	−0.005	–	–	0.626	0.992
E	–	0.015	0.242	–	−0.006	–	–	0.592	0.990
F	–	–	0.310	–	−0.014	–	–	–	0.983
G	–	–	0.318	–	–	–	–	–	0.885
Mean									0.96 ± 0.04

## Discussion

The present study investigated the extent to which pitch location changes when the release parameters vary within a realistic range. By comparing the fluctuation of the simulated pitch location when varying each release parameter, it was found that the elevation pitching angle and speed significantly influenced the vertical pitch location, and the azimuth pitching angle significantly influenced the horizontal pitch location. Moreover, a regression model was obtained to predict the pitch location, and it became clear that the significant predictors for the vertical pitch location were the elevation pitching angle, speed, and spin axis, and those for the horizontal pitch location were the azimuth pitching angle, spin axis, and horizontal release point. Therefore, it was suggested that the parameter most affecting pitch location was pitching angle.

It can be considered that the method used in this study identifies the variability of the pitch location when each release parameter fluctuates from the average value of each pitcher. However, how easily the release parameters themselves vary may be different. Therefore, here, by interpreting the fluctuation range of the measured value of each release parameter as the easiness of variation of each release parameter, the influence of each release parameter on the pitch location was compared as fairly as possible.

Based on the above, it can be considered that the variation in the pitch location is mainly caused by the variation in the pitching angle, suggesting that adjustment of the pitching angle is a crucial factor for accuracy of baseball pitching. By comparing the pitch location when each parameter was varied independently, it was found that the pitching angle and speed, i.e., the velocity vector, significantly affected the pitch location for all pitchers. In particularly, with respect to pitching angle, it was found that a deviation of several degrees produces a deviation of several tens of centimeters. This result was further supported by multiple regression analysis. The reason why such a result was obtained may be that pitching is a task for which the speed of the projectile at the time of release is relatively large compared to the other throwing tasks, and the distance to the target is sufficiently long. It is considered that the variation in the pitching angle, which is the direction of the velocity vector, greatly affected the variation in the pitch location, the distance to which is long. Moreover, how release parameters are defined also the one of the reasons. However, the release parameters used in this study are common in expressing the ball movement. In previous studies, the same way to define the release parameters as this study (ex. Nagami et al., 2011).

### Relationship Between Release Parameters and Difference Between Pitchers

Although the fluctuation of the spin axis was very small in the simulation, its explanatory rate for pitch location was high for many pitchers. This result showed that spin axis had little influence when it was fluctuated independently but had a significant explanatory rate when combined with other parameters. Therefore, it was suggested that the spin axis covariated with other parameters to affect pitch location. The release point was conversely shown to cause fluctuation of pitch location in the simulation, but it had no explanatory rate for pitch location. This result suggested that the influence of release point was canceled by changes of other parameters. Thus, the release point may have a cooperative relationship with other parameters. Some parameters, such as spin rate, showed little influence on the variability of pitch location in both the simulation and regression analysis. However, in the previous study, the ratio of the spin rate to ball speed considering the direction of spin axis (i.e., effective spin parameter) would affect the lift coefficient more strongly than the spin rate and the spin axis separately (Nagami et al., 2016). Therefore, it was suggested that even for parameters that did not show a significant effect independently, certain combination of parameters may affect pitch location more strongly than individual parameters. In summary, these results mean that it is important that the influence of each release parameter and the combination of parameters on pitch location are considered separately.

In addition to the similar trend among all pitchers in the simulation analysis, the multiple regression analysis showed that the explanatory rates of each parameter were different for each pitcher. This indicates that the elevation pitching angle and speed are common factors in determining pitch location, but other parameters, such as the spin axis and release point, likely have different relations among the release parameters for each pitcher. One of the reasons for this difference might be that there can be specific combinations of release parameters in individual pitchers, even when targeting the same location (Jinji and Sakurai, 2006; Nagami et al., 2011). Because 9 parameters are related to pitch location in baseball pitching, there are countless combinations of values of release parameters that achieve the same pitch location. Therefore, the pitcher can select various values of release parameters. It is clear that the best method for realizing throwing accuracy is not necessarily improving the reproducibility of each parameter, as in previous studies that used two-dimensional motions (Kudo et al., 2000; Cohen and Sternad, 2009; Nasu et al., 2014). The same interpretation could be applied to baseball pitching, which is more dynamic and has more degrees of freedom. Future research will investigate how to control the effect of the parameters, especially pitching angle that was found to have a large effect on the pitch location in this study. If future studies investigate the relationship between parameters and how to adjust them in more detail, it might become possible to better understand the mechanism of accuracy in baseball pitching. The results of this study contribute to understanding accuracy in various sports-related motor skills for which many parameters are complicatedly related in terms of extracting important components.

### Limitations of This Study

Some limitations of this study should be noted. The experimental environment in this study was different from that in actual baseball games. The data was measured indoors so as to reduce the effect of wind and air currents on the aerodynamic characteristics of the ball that affect the ball trajectory. The participants always threw at the same target position, and there was no batter. Some release parameters might be influenced by these factors. However, the main results of study are considered not to be different largely by these factors because the release parameters used in this study did not have great difference from the studies have referred before. Moreover, sample size of this study was small. Only seven skilled baseball pitchers participated in the study. Different results may be obtained with more participants with various skill levels. It may need to investigate more participants in more practical settings in the future study.

It should be noted that the result may be specific to the fastball used in this study. The participants threw only 4 seam fastballs in this study, but pitchers throw various types of pitches, such as braking balls, in actual baseball games. Previous studies investigating release parameters for various ball types have shown that, depending on the type of ball, the spin rate and the spin axis can significantly influence on the trajectory of the ball (Jinji and Sakurai, 2006; Nagami et al., 2016). Considering this point, depending on the type of the ball, it is possible that other parameters such as spin rate and spin axis in addition to the pitching angle may affect the pitch location. Moreover, the seams of the ball ware not taken into account in the ball mechanics simulation. The seams may require consideration when breaking balls are investigated.

### Application in Actual Fields

This is the first study to investigate the influence of release parameters including spin parameters, on the pitch location. The fluctuation of the pitch location was simulated for variation of each release parameter, and it was revealed that each parameter's contribution to the pitching accuracy varied. In previous studies, the pitch location was found to be related to variability in joint kinematics and ball release timing (Hore, 1996; Timmann et al., 1999; Fleisig et al., 2009), whereas the release parameters have not been studied thoroughly. The flight trajectory and pitch location were finally determined by the dynamical state at the time of release as a result of movements of body. This study furthered our knowledge of release parameters that connect body movements and pitch location and have a deterministic influence on pitching accuracy.

With recent advances in science and technology, measurement equipment, and data analysis technology have made remarkable progress. For example, TrackMan Baseball (TrackMan, Denmark), which was developed in 2003, is generally used as a data analysis system in major league baseball. Because it is possible to easily measure various parameters of the ball with high precision and in real time, practice, and teaching can be aimed at measurable numerical values rather than ambiguous feeling. From this research, we were able to show that the contribution to pitching accuracy varies depending on the parameters. As it is difficult to be conscious of multiple aspects during actual movements, extracting important elements may be useful for practice and teaching. Moreover, understanding the differences of individuals may contribute to performance improvements. Pitching involves multiple skills, such as increasing the ball speed, improving control, and learning breaking balls. Therefore, various styles can coexist even among skilled pitchers. When targeting tasks as pitching, it is important for coaches and players not only to know the tendency seen among skilled players and but to pay attention to cases that deviate from it. The advantage of the approach in this study with the incorporation of theoretical knowledge of body and ball movements, is that the knowledge about what is typical and when it may not be valid is acquired.

## Conclusion

This study revealed the degree of influence of each release parameter on the pitch location in baseball pitching. The fluctuation of the pitch location was simulated for variation of each release parameter. It was revealed that, the elevation pitching angle and speed significantly influenced the vertical pitch location, and the azimuth pitching angle significantly influenced the horizontal pitch location. Moreover, a regression model was obtained to predict the pitch location, and it became clear that the significant predictors for the vertical pitch location were the elevation pitching angle, speed, and spin axis (θ3), and those for the horizontal pitch location were the azimuth pitching angle, spin axis, and horizontal release point. Therefore, it was suggested that the parameters most affecting pitch location were pitching angle. In future work, we will consider a relationship of parameters more clearly to further elucidate the factors affecting pitch location.

## Data Availability Statement

The datasets generated for this study are available on request to the corresponding author.

## Ethics Statement

The studies involving human participants were reviewed and approved by the ethics committee of the University of Tokyo. The patients/participants provided their written informed consent to participate in this study.

## Author Contributions

AK, KK, KN, and SW contributed conception and design of the study. HK, TM, and MK performed experiments. AK performed the analysis and wrote the first draft of the manuscript. KN revised partially the manuscript. All authors contributed to manuscript revision, and read and approved the submitted version.

### Conflict of Interest

The authors declare that the research was conducted in the absence of any commercial or financial relationships that could be construed as a potential conflict of interest.
