# Supplementary material for: Influence of Release Parameters on Pitch Location in Skilled Baseball Pitching
Source: Front Sports Act Living. 2020 Apr 15;2:36. doi: 10.3389/fspor.2020.00036 (PMC7739723; doi:10.3389/fspor.2020.00036)
Supplement: Supplementary file 1 [file Table_1.DOCX]

Table 1 Variation of vertical pitch location (cm) when varied each parameter from the minimum to the maximum values.

| Pitchers | v | θ₁ | θ₂ | n | θ₃ | x | y | z |
| --- | --- | --- | --- | --- | --- | --- | --- | --- |
| A | 36.9 | 117.0 | 0.9 | 1.7 | 2.5 | 2.2 | 10.0 | 0 |
| B | 12.2 | 102.0 | 0.4 | 1.6 | 4.1 | 3.1 | 13.3 | 0 |
| C | 8.2 | 72.8 | 0.4 | 0.4 | 2.3 | 1.5 | 9.2 | 0 |
| D | 24.6 | 102.0 | 0.3 | 0.9 | 3.6 | 1.9 | 4.2 | 0 |
| E | 9.5 | 192.0 | 0.8 | 4.4 | 2.5 | 1.4 | 6.3 | 0 |
| F | 15.5 | 202.8 | 0.5 | 1.4 | 2.2 | 1.9 | 3.8 | 0 |
| G | 17.5 | 86.8 | 0.7 | 1.5 | 1.5 | 2.3 | 7.1 | 0 |
|  | 17.8 ± 9.3 | 125.1 ± 47.6 | 1.7 ± 1.2 | 1.8 ± 1.4 | 2.7 ± 0.8 | 2.0 ± 0.5 | 7.7 ± 3.1 | 0 |

Table 2 Variation of horizontal pitch location (cm) when varied each parameter from the minimum to the maximum values.

| Pitchers | v | θ₁ | θ₂ | n | θ₃ | x | y | z |
| --- | --- | --- | --- | --- | --- | --- | --- | --- |
| A | 1.4 | 0.1 | 133.0 | 3.5 | 0.7 | 1.4 | 0 | 19.4 |
| B | 0.3 | 0.6 | 59.0 | 1.0 | 1.0 | 2.5 | 0 | 9.7 |
| C | 0.1 | 0.5 | 58.9 | 0.2 | 4.4 | 0.4 | 0 | 8.9 |
| D | 0.9 | 0.5 | 58.8 | 1.0 | 3.4 | 0.7 | 0 | 11.0 |
| E | 0.4 | 0.4 | 103.0 | 1.5 | 1.4 | 0.5 | 0 | 12.1 |
| F | 0.2 | 2.3 | 72.5 | 0.2 | 10.6 | 0.3 | 0 | 8.0 |
| G | 0.1 | 0.8 | 117.0 | 0.4 | 6.5 | 1.0 | 0 | 12.0 |
|  | 0.5 ± 0.4 | 0.7 ± 0.7 | 86.0 ± 28.9 | 1.1 ± 1.1 | 4.4 ± 3.3 | 1.0 ± 0.7 | 0 | 11.6 ± 3.5 |

Table 3 Coefficient of regression equation for each pitcher

| Pitcher | a | b | c | d | e | f |
| --- | --- | --- | --- | --- | --- | --- |
| A | -8.07 | 0.424 | 0.288 | -0.00524 | 0.00015 | -0.00037 |
| B | -8.57 | 0.485 | 0.284 | -0.00629 | 0.00024 | -0.00036 |
| C | -8.67 | 0.469 | 0.281 | -0.00590 | 0.00031 | -0.00041 |
| D | -6.44 | 0.332 | 0.287 | -0.0383 | 0.00031 | -0.00042 |
| E | -5.23 | 0.256 | 0.256 | -0.00269 | 0.00001 | -0.00031 |
| F | -6.35 | 0.317 | 0.289 | -0.00363 | 0.00006 | -0.00033 |
| G | -8.72 | 0.486 | 0.286 | -0.00639 | 0.00017 | -0.00043 |
